# Elucidating the Morphology of the Endoplasmic Reticulum: Puzzles and Perspectives

**DOI:** 10.1021/acsnano.3c01338

**Published:** 2023-06-28

**Authors:** Reinhard Lipowsky, Shreya Pramanik, Amelie S. Benk, Miroslaw Tarnawski, Joachim P. Spatz, Rumiana Dimova

**Affiliations:** † Max Planck Institute of Colloids and Interfaces, 14424 Potsdam, Germany; ‡ Max Planck Institute for Medical Research, 69120 Heidelberg, Germany

**Keywords:** membrane morphology, endoplasmic reticulum, membrane elasticity, Steiner minimal trees, triunduloids, curvature elasticity, membrane fission

## Abstract

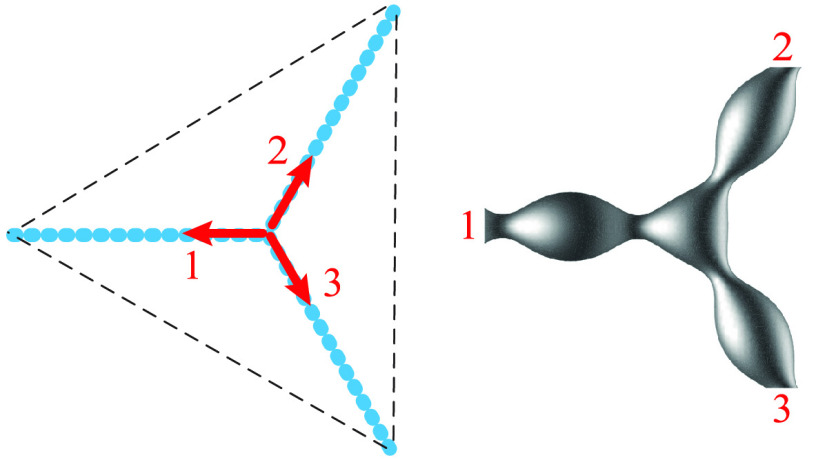

Artificial or synthetic organelles are a key challenge for bottom-up synthetic biology. So far, synthetic organelles have typically been based on spherical membrane compartments, used to spatially confine selected chemical reactions. In vivo, these compartments are often far from being spherical and can exhibit rather complex architectures. A particularly fascinating example is provided by the endoplasmic reticulum (ER), which extends throughout the whole cell by forming a continuous network of membrane nanotubes connected by three-way junctions. The nanotubes have a typical diameter of between 50 and 100 nm. In spite of much experimental progress, several fundamental aspects of the ER morphology remain elusive. A long-standing puzzle is the straight appearance of the tubules in the light microscope, which form irregular polygons with contact angles close to 120°. Another puzzling aspect is the nanoscopic shapes of the tubules and junctions, for which very different images have been obtained by electron microcopy and structured illumination microscopy. Furthermore, both the formation and maintenance of the reticular networks require GTP and GTP-hydrolyzing membrane proteins. In fact, the networks are destroyed by the fragmentation of nanotubes when the supply of GTP is interrupted. Here, it is argued that all of these puzzling observations are intimately related to each other and to the dimerization of two membrane proteins anchored to the same membrane. So far, the functional significance of this dimerization process remained elusive and, thus, seemed to waste a lot of GTP. However, this process can generate an effective membrane tension that stabilizes the irregular polygonal geometry of the reticular networks and prevents the fragmentation of their tubules, thereby maintaining the integrity of the ER. By incorporating the GTP-hydrolyzing membrane proteins into giant unilamellar vesicles, the effective membrane tension will become accessible to systematic experimental studies.

## Introduction

The bottom-up approach to synthetic biology has the long-term objective of creating artificial cells by the assembly of synthetic modules. One important module is provided by synthetic membrane compartments mimicking the biological membranes around cells and organelles.^1−3^ Each eukaryotic cell is subdivided into many membrane-bound compartments or organelles that provide the spatial separation of different cellular processes and functions. Therefore, a key challenge for synthetic biology is assembling artificial organelles that perform some of these cellular functions. One important objective that has been recently achieved to some extent is the confinement of selected catalytic or enzymatic reactions within spherical liposomes and polymersomes.^4−9^ However, the morphology of membrane-bound organelles in cells is often far from spherical and can exhibit a fairly complex architecture. A particularly fascinating example is provided by the nanotubular networks of the endoplasmic reticulum (ER).

**Figure 1:**
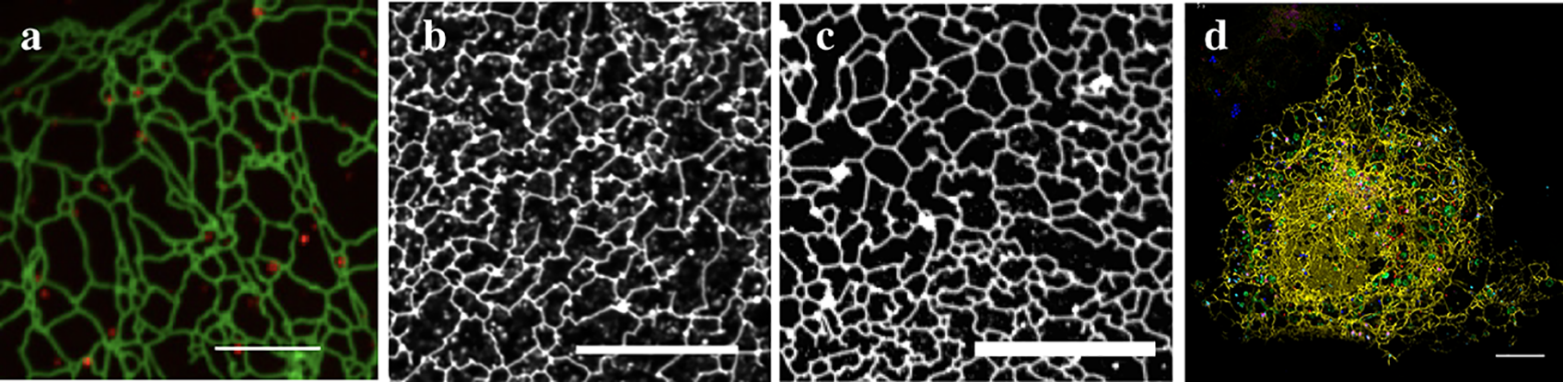
Irregular polygonal networks of nanotubes with three-way junctions: (a) ER network as observed by live cell imaging with tubules labeled by a green fluorescent protein. Scale bar: 5 μm. Reprinted with permission from ref (16) (Copyright 2013, Friedman et al.). (b,c) Reticular networks reconstituted from proteoliposomes containing (b) two membrane proteins, Yop1p and Sey1p, from yeast as well as (c) a single membrane protein, atlastin from *Drosophila melanogaster* (DmATL). Scale bars: 20 μm. Reprinted with permission from ref (25) (Copyright 2017, Springer Nature). (d) Three-dimensional extension of the ER network visualized by advanced fluorescence microscopy. The ER tubules (yellow) interact with other organelles such as mitochondria (green), peroxisomes (red), and Golgi (magenta) via membrane contact sites. Scale bar: 10 μm. Reprinted with permission from ref (18) (Copyright 2017, Springer Nature).

The ER is the largest membrane-bound organelle that is present in all eukaryotic cells. Each cell has only a single copy of this organelle, which contains, however, more than half of the total cellular membrane.^10^ Based on the intensity of the fluorescently labeled ER membrane, one can distinguish different ER subregions that also differ in the dominant morphologies formed by the membrane.^11^ In the nuclear envelope and in the perinuclear subregion close to the nucleus, the ER membrane forms many sheets, whereas it forms narrow nanotubes in the peripheral subregion further away from the nucleus. The membrane nanotubes are interconnected by junctions and form a continuous network that extends throughout the whole cell.^11−18^ The large majority of these junctions is three-way junctions, at which three tubules meet (Figure 1a–c).^14^ Even though the connectivity of the ER network is primarily based on such three-way junctions, this network is truly three-dimensional (Figure 1d).^11,18^ The latter image displays many junctions that are stacked on top of each other and are thus difficult to resolve individually, apart from those at the periphery.

The ER nanotubes have a typical diameter between 50 and 100 nm^11,19,20^ and are visible in Figure 1 because the tubular membranes are fluorescently labeled. The high curvature of the nanotubes is generated by two evolutionarily conserved protein families, the reticulons and receptor expression enhancing proteins (REEPs) such as Yop1p in yeast.^21,22^ In the context of curvature elasticity, the formation of nanotubes implies a large spontaneous curvature, which is comparable to the inverse diameter of the tubes.^23,24^

In spite of its complex architecture, the whole nanotubular network of the ER is formed by a single membrane that encloses a continuous nanofluidic network of water channels.^11,26,27^ The continuity of the ER lumen has been demonstrated by monitoring the diffusion of fluorescently labeled molecules, using a variety of experimental techniques such as fluorescence recovery^26^ and single particle tracking.^11,27^ As a consequence, the ER membrane creates a bicontinuous structure that partitions the intracellular space into two separate, interpenetrating subcompartments: the lumen of the ER network and the surrounding cytosol. In fact, the ER membrane itself provides two additional quasi-two-dimensional subcompartments corresponding to the two leaflets of the lipid–protein bilayer, which can accommodate membrane-bound molecules and processes. All four subcompartments are liquid (or fluid), which implies that widely separated regions of the cell can communicate with each other, both via the ER membrane and via the ER lumen. Furthermore, this architecture implies that the ER forms a membrane surface with a large area-to-volume ratio and with a very high topological genus,^28^ arising from the many irregular polygons formed by the nanotubes. Bicontinuous structures have also been studied for microemulsions of oil–water–surfactant mixtures^29^ and for mesophases phases^30^ of lipid–water systems.

For some time, it was thought that the formation of reticular networks requires the presence of microtubules and cytoskeletal motors.^14,15,31^ However, in vitro reconstitution experiments based on cell extracts^32^ and on proteoliposomes^25,33−36^ have demonstrated that the formation of such networks does not require cytoskeletal components. On the other hand, in order to generate a reticular network, the reconstituted systems must contain a membrane protein that is able to hydrolyze GTP.^25,33,34,37,38^ In Figure 1b,c, this membrane protein is provided by the yeast protein Sey1p and by the atlastin protein from *Drosophila melanogaster* (DmATL), respectively.^25^

Reticular networks with three-way junctions are formed by membrane fusion,^14^ which is coupled to GTP hydrolysis and mediated by the *trans*-dimerization of membrane GTPases such as atlastin.^33,34,39^ Furthermore, the synthetic reticular networks displayed in Figure 1b,c were obtained for a low density of membrane-bound protein. The network in Figure 1c, for example, which involves only the membrane GTPase atlastin, was prepared with a protein-to-lipid ratio of 1:1000.^25^ As a consequence, the membrane-bound atlastins were well separated from each other with an average separation of at least 19 nm, corresponding to a dilute regime without crowding.

We will focus here on the simplest reticular membrane networks as reconstituted from lipids and one or two membrane proteins. These in vitro networks form irregular polygonal networks that look very similar to the peripheral networks observed in vivo; see Figure 1. Recent studies using superresolution light microscopy^17^ and three-dimensional electron microscopy^40^ revealed dense clusters of three-way junctions, so-called ER matrices, which have been modeled as periodic networks built up from unit cells with hexagonal symmetry.^41^ However, these ER matrices have not been reconstituted in vitro^11^ and, thus, will not be addressed here.

The ER membrane also forms sheets consisting of essentially flat membrane segments, connected by highly curved segments along the sheet edges. Different sheet morphologies have been distinguished, including stacked helicoidal sheets, twisted sheets, ER cisternae, and fenestrated sheets.^11^ The edges of ER sheets are stabilized by curvature-generating membrane proteins, similar to those found in ER tubules, whereas the stabilization of the flat sheet segments involves other membrane proteins such as Climp63, p180, and KTN (or kinectin).^11,42−45^ The mechanisms underlying the sheet stabilization by p180 and KTN are not clear, but Climp63 is thought to form bridges across the lumen of the ER sheets, thereby maintaining the nearly constant spatial separation of the two flat membrane segments. So far, the morphological diversity and molecular complexity of ER sheets have precluded their in vitro reconstitution.

Our paper is organized as follows. First, we explain the observed predominance of three-way junctions in terms of Steiner minimal trees, a concept borrowed from mathematical graph theory, and argue that this predominance provides strong evidence of a substantial tension of the ER membrane on the micrometer scale. Second, we consider the nanoscopic shapes of tubes and junctions that resemble the constant-mean-curvature shapes of unduloids and triunduloids. The latter shapes have very low bending energies when their mean curvature is close to that of the spontaneous membrane curvature. When the membrane necks of unduloids and triunduloids become closed, these necks can be cleaved, which leads to the fragmentation of the tubular networks as observed in vitro.^25^ Such fragmentation is avoided by a sufficiently large membrane tension that prevents the necks from closing. Third, we argue that such a tension can be generated by the *cis*-dimerization of atlastin and other GTP-hydrolyzing membrane proteins. Finally, we describe future experimental studies to elucidate the proposed mechanism.

## Results and Discussion

### Three-Way Junctions of Reticular Networks

The geometry of the reticular networks, as observed in the fluorescence microscope (Figure 1) exhibits some intriguing features. The tubules and junctions form irregular polygons that are bounded by straight tube segments and have interior angles close to 120°. Likewise, the contact angles between the tubules that form at a three-way junction are close to 120° as well. These geometric features have already been observed in early light microscopy studies of the ER,^12−14^ but the underlying mechanism has remained elusive.

#### Force Balance at Junctions

**Figure 2:**
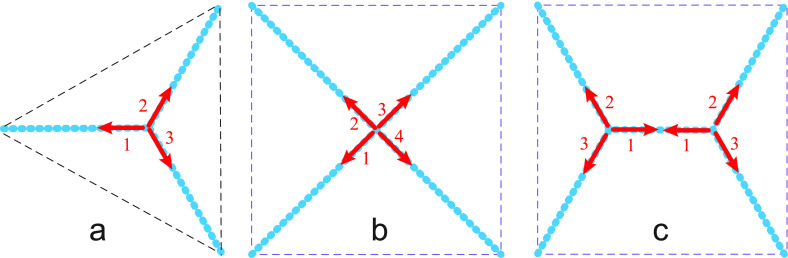
Three-way and four-way junctions of the membrane nanotubes. Because the tubules (blue) are fluid, force balance at such a junction implies that the force vectors (red) arising from the membrane tension balance each other and that all contact angles between neighboring tubules have the same value. (a) Three-way junction with three force vectors 1, 2, and 3 and three contact angles of 120°. (b) Four-way junction with four force vectors 1, 2, 3, and 4 and four contact angles of 90°. (c) Two three-way junctions, each with three force vectors and three contact angles of 120°. The total length of the tubules in (b) is larger than the total length of the tubules in (c), which implies a reduction in the elastic membrane energy when we transform the four-way junction in (b) into two three-way junctions, as in (c).

These basic geometric features can be understood if the tubular membranes experience a significant membrane tension. In the absence of such a tension, membrane tubes undergo strong shape fluctuations that move the tubes in and out of the focal plane of the optical microscope,^46^ which would necessarily lead to deformed tube shapes and to blurred images in Figure 1. Furthermore, such a membrane tension acts to minimize the total length of the nanotubes. The fluidity of the membranes then implies that all tubules that meet at a junction must experience the same tension.^47^ If we now consider three nanotubes that meet at a three-way junction, the nanotubes will exert three forces onto the junction, and the corresponding force vectors will balance each other (Figure 2a). As a consequence, the three tubes are located within the same plane and form contact angles of 120°.

Now, let us extend these considerations to a four-way junction at which four tubules meet. Force balance now implies that the contact angle between two neighboring tubules is 90° (Figure 2b). The membrane tension contributes an elastic energy that is proportional to the total length of the junctional arms. Because the membrane is fluid, the four-way junction can be divided up into two three-way junctions, each of which has contact angles of 120° (Figure 2c). A simple computation shows that the total length of the tubules with two three-way junctions is smaller than the total length of the four-way junction, which implies that the mechanical membrane tension and the membrane’s elastic energy are reduced when we transform the four-way junction into two three-way junctions. This transformation is intimately related to Steiner minimal trees^48−53^ as studied in mathematical graph theory.

#### Networks in Three Dimensions

The networks displayed in Figure 2 are two-dimensional. However, Steiner minimal trees can also be constructed for three-dimensional networks.^50,51,53^ A simple example is provided by the regular tetrahedron, for which the four corner vertices are connected by six edges of equal length. These edges can be grouped into three pairs of mutually orthogonal edges. Furthermore, each pair of orthogonal edges defines a “diagonal line” that goes through the two midpoints of the two orthogonal edges. Now, let us add a fourth vertex at the centroid of the tetrahedron and connect this vertex to all four corner vertices of this tetrahedron, thereby creating a four-way junction in three dimensions, in analogy to the two-dimensional case in Figure 2b. The total length of the connecting edges can again be reduced by splitting this four-way junction into two three-way junctions and moving these two junctions apart from each other along one of the three “diagonal lines” of the regular tetrahedron, in analogy to Figure 2c. The main difference to the latter figure is that the two three-way junctions arising from the centroid of the tetrahedron are no longer located in the same plane but are now rotated against each other by 90°.

Therefore, the intriguing architecture of the reticular networks (Figure 1) indicates that the ER membrane is subject to significant membrane tension on the microscale. What remains to be understood is the origin of this tension. Further below, we will argue that this tension is generated by GTP hydrolysis coupled to cis-dimers of two membrane proteins in the same membrane.

### Nanoscopic Shapes of Tubules and Junctions

#### Experimental Observations

**Figure 3:**
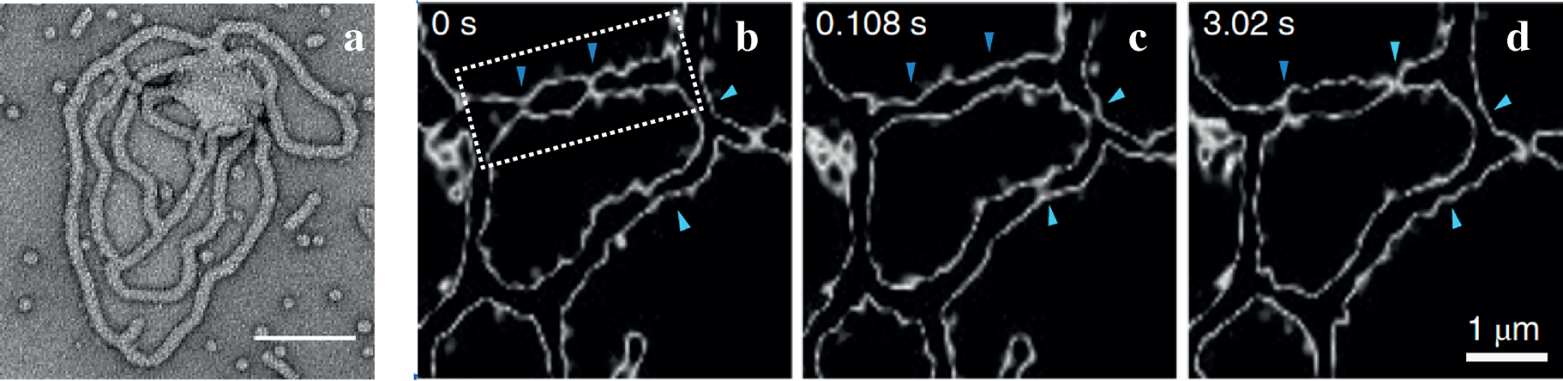
Shapes of membrane nanotubes and junctions: (a) Electron microscopy image of a reconstituted network with apparently cylindrical tubes. Scale bar: 100 nm. Reprinted with permission from ref (25) (Copyright 2017, Springer Nature). (b–d) Three time-lapse images of an in vivo network with undulating tubules and transient membrane necks (blue arrowheads) as obtained by structured illumination microscopy. The time-lapse images display the shape changes of an irregular pentagon consisting of five three-way junctions connected by five tubular segments. The third arm of each junction is only partially visible and provides a connection to another junction outside the field of view. The four blue arrow heads point to membrane necks (constrictions) formed by three tubular segments, which resemble peristaltic shape deformations of these tubules. The tube segment (TS1) within the white dotted rectangle in (b) has a length of about 2.5 μm and an average diameter of about 200 nm. Time points in seconds (upper left corners); scale bar in (d) also applies to parts (b) and (c). Reprinted with permission from ref (27) (Copyright 2018, The Authors).

Another puzzling aspect of the ER morphology is the shapes of the tubules and junctions at the nanoscale. Indeed, very different images of these nanoscopic shapes have been obtained by electron microscopy (EM) and structured illumination microscopy (SIM). Negative-stain EM images (Figure 3a) are consistent with cylindrical tubes that form junctions with sharp kinks.^25^ In contrast, time-lapse images obtained by SIM show undulating tubules (Figure 3b–d) with mobile constrictions or open membrane necks (blue arrows).^27^ Both imaging methods have their drawbacks. Negative-stain EM is a rather harsh technique and likely to deform the nanotubes.^25^ Indeed, a comparison of the EM image in Figure 3a with the images in Figure 1b,c as obtained by confocal fluorescence microscopy shows that only a small part of the network has survived the harsh treatment by negative EM staining and that the mesh size of the network has been strongly reduced.

Compared to EM, SIM is a fairly new experimental technique of superresolution light microscopy that has a poorer resolution of about 100 nm.^54,55^ This resolution limit should be compared to the size of the open membrane necks formed by the tubules (Figure 3b–d, arrowheads), some of which are below the resolution limit. In addition, the average diameter of the tubules in Figure 3b–d is about 200 nm, which is relatively large. Thus, the details of the undulating shapes in Figure 3b–d are somewhat uncertain, but undulating (or oscillating) shapes of ER tubules have also been observed by other groups.^11,17^ In any case, the nanotubular junctions should not form sharp kinks, as in the EM image of Figure 3a, because such kinks represent highly curved membrane segments that would lead to a very large bending energy of the junctions.

#### Networks Based on Unduloids and Triunduloids

**Figure 4:**
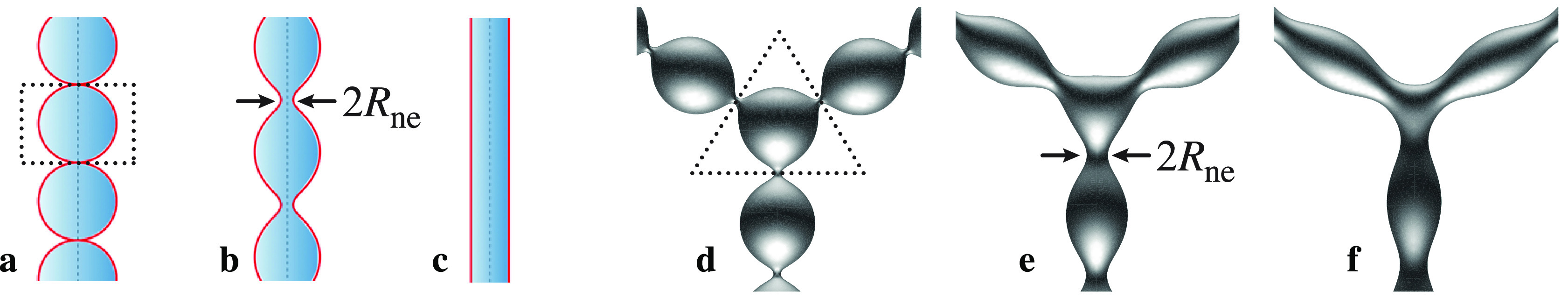
Six different membrane shapes with the same constant mean curvature *M*, distinguished by different radii *R*_ne_ of their membrane necks: (a–c) Unduloids which are tubules with membrane necks that are placed periodically along the axis of rotational symmetry.^57,58^ The neck radius varies from *MR*_ne_ = 0 for the multispherical shape in (a) to *MR*_ne_ = 1/2 for the cylindrical shape in (c). The unduloid in (b) represents a peristaltic shape deformation of the cylinder in (c). (d–f) Triunduloids with three unduloidal arms and 120° contact angles,^62^ providing smoothly curved membrane shapes for the three-way junctions in Figures 1 and 3. Each triunduloid has a central core segment connected to three unduloids by three membrane necks. The neck radius *R*_ne_ varies from *MR*_ne_ = 0 in (d) to *MR*_ne_ = 1/3 in (f), thereby excluding a triunduloid connecting three cylindrical tubes. Reprinted with permission from ref (62) (Copyright 1997, Springer-Verlag).

In contrast, *non*-cylindrical shapes of tubes and junctions, which resemble unduloids and triunduloids as in Figure 4, have very low bending energies.^56^ Both unduloids and triunduloids represent surfaces of constant mean curvature, as studied in differential geometry. Unduloids as in Figure 4a–c were originally obtained by Delaunay in 1841,^57,58^ whereas the triunduloids displayed in Figure 4d–f were not constructed until the early 1990s.^59−63^ Unduloids can be viewed as deformed cylinders with periodically placed membrane necks of radius *R*_ne_. Triunduloids consist of three unduloids that are connected to a central core membrane segment via three membrane necks, thereby forming a three-way junction. The membrane necks can be open as in Figure 4b,c,e,f or may become closed as in Figure 4a,d.

All unduloids and triunduloids displayed in Figure 4 have the same constant mean curvature denoted by *M*. The neck radius *R*_ne_ of the unduloids in Figure 4a–c can vary between *R*_ne_ = 0, which applies to the multispherical chain of equally sized spheres with radius *R*_sp_ = 1/*M* in Figure 4a,^56^ and *R*_ne_ = 1/(2*M*), which represents a cylinder with radius *R*_cy_ = 1/(2*M*). On the other hand, for the triunduloids in Figure 4d–f, the neck radius *R*_ne_ can only vary within a reduced range, between *R*_ne_ = 0, which again corresponds to a multispherical shape consisting of equally sized spheres with radius *R*_sp_ = 1/*M*, and *R*_ne_ = 1/(3*M*), as displayed in Figure 4f.^59,62^ Therefore, it is not possible to construct a triunduloid by connecting three cylinders as in Figure 4c to a central core segment.

The bending energy of the membrane depends on the deviation of mean curvature *M* from spontaneous (or preferred) membrane curvature *m*. In fact, each constant-mean-curvature shape with *M* = *m* has vanishing bending energy and provides a global minimum of the bending energy.^56^ Therefore, all unduloids and triunduloids in Figure 4 have a low bending energy when their constant mean curvature is close to the spontanoeus curvature *m*. As a consequence, a single irregular polygon of the ER network is expected to exhibit many metastable states with similar energy levels. When the tubular membranes of such a polygon undergo shape fluctuations on smaller length scales, arising from thermal noise or driven by conformational changes of membrane-bound proteins, their shapes will become rather similar to the SIM images in Figure 3b–d, where membrane necks (constrictions) are indicated by blue arrowheads. Inspection of the corresponding time-lapse movie^27^ shows that the closed membrane necks in Figure 3b–d represent transient membrane shapes that reopen again, corresponding to peristaltic shape fluctuations of the tubules.

#### Closed Membrane Necks and Nanotube Fragmentation

Closed membrane necks as in Figure 4a,d represent special membrane segments of a nanotubular network that are most likely to undergo membrane fission. Indeed, such a fission process must overcome a free energy barrier arising from the local disruption of the lipid–protein bilayer. On the supramolecular scale, one may envisage this barrier to arise from a cut across the bilayer and the creation of two ring-like hydrophobic bilayer edges.^47,64^ The mechanical work of fission is proportional to the total length of the bilayer edges. This length is minimal if the nanotube is cleaved across a closed membrane neck. Furthermore, neck cleavage is facilitated when the membrane has a large spontaneous curvature as demonstrated experimentally by the controlled division of giant vesicles via low densities of membrane-bound proteins.^65^

Therefore, if an open membrane neck in Figures 3b–d or 4b,e became closed for an extended period of time, the neck could undergo fission, thereby breaking the nanotubes up into several fragments. For reticular networks, such a fragmentation of the nanotubes has indeed been observed experimentally, both in vivo and in vitro, by downregulating the biosynthesis of GTP-hydrolyzing membrane proteins^33^ and by interrupting the supply of GTP.^25^ As a consequence, any mechanism that keeps the membrane necks open also prevents nanotube fragmentation.

#### Fragmentation of Tubes via Peristaltic Modes

To obtain additional insight into the fragmentation of membrane nanotubes, the shape fluctuations of the tubular segments in the reticular network are decomposed into Fourier modes. We will focus on peristaltic modes, which turn out to be the most unstable modes. The fluctuation spectrum of these modes is described in the Methods section, where the effective membrane tension Σ is used to control the excess area stored in the shape fluctuations. For a cylindrical tube with radius *r*_0_ and length *L*, the most unstable mode, denoted by , is characterized by wavenumber *p* = p_*_ = 1/*r*_0_, period 2*πr*_0_, and mean-squared amplitude
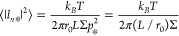
1as follows from eq 9 in the Methods section. The amplitude  as given by eq 1 grows as 1/Σ for small Σ. Thus, the tube should become fragmented for sufficiently small tensions Σ ≤ Σ_1_, for which the amplitude of the most unstable mode exceeds the tube radius, that is, for
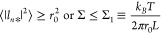
2

The tension threshold Σ_1_ is very low. As an example, consider the TS1 tube segment within the dotted white rectangle in Figure 3b, which has a length of *L* = 2.5 μm and a radius of *r*_0_ = 100 nm. For this tube, the tension Σ_1_ has a value of 4 × 10^–6^ mN/m at room temperature.

In Figure 3b–d, the peristaltic modes have smaller amplitudes that do not lead to tube fragmentation. Inspection of this figure indicates that the most unstable modes have amplitudes of about half the cylinder radius, which define another tension Σ_2_ via
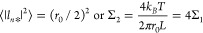
3As shown in the Methods section, the average excess area  stored in the shape fluctuations of the tube is reduced from about 10% of the tube area for Σ = Σ_1_ to about 5% of the tube area for Σ = Σ_2_.

### GTP Hydrolysis and Protein Dimerization

#### Network Formation and GTP Hydrolysis

The interdependence of network formation and GTP hydrolysis was originally observed for a cytosolic medium that did not allow to identify the protein that was responsible for the hydrolysis.^32^ The missing protein was later identified to be the membane protein atlastin,^33,34^ which mediates GTP-dependent membrane fusion. The fusion of atlastin-containing proteoliposomes was demonstrated both by lipid mixing^33^ and by content mixing,^37^ the two standard fusion assays based on fluorescence resonance energy transfer. Lipid mixing provides direct evidence for the exchange of lipids between the outer leaflets of the two membranes to be fused, whereas content mixing demonstrates the exchange of aqueous solution between the interior compartments of the two vesicles and, thus, the formation of a fusion pore across the two adjacent bilayer membranes. Atlastin from *Drosophila melanogaster* (DmATL) and other proteins from the atlastin family mediate GTP-dependent membrane fusion in the ER of multicellular animals, while analogous membrane proteins such as Seyp1 and RHD3 catalyze membrane fusion in yeast^66^ and plant^67^ cells.

#### GTP Hydrolysis Coupled to *trans*- and *cis*-Dimerization

**Figure 5:**
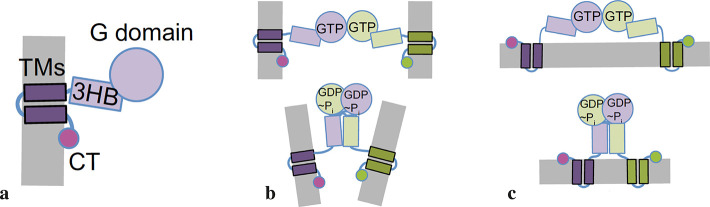
Model for active dimerization of DmATL: (a) DmATL protein (purple) anchored to a lipid membrane (gray). The protein consists of several domains that are distinguished by different shades of purple: the GTP-hydrolyzing (G) domain, which binds and hydrolyzes GTP; the three-helix-bundle (3HB) domain; the two transmembrane (TM) domains; and the cytosolic tail (CT) domain. (b) *trans*-Dimerization of two atlastin molecules anchored in two different lipid membranes, and (c) *cis*-dimerization of two atlastin molecules anchored in the same membrane. In both (b) and (c), the upper cartoon displays the initial dimerization via the two G domains, both of which are loaded with GTP, while the lower cartoon depicts the crossover dimerization of the G and 3HB domains, which occurs after GTP has been cleaved to guanosine diphosphate (GDP) and inorganic phosphate (P_i_). During crossover dimerization, the two G domains swap their positions, and the two 3HB domains come into contact. Reprinted with permission from ref (38) (Copyright 2015, PNAS).

The different protein domains of membrane-bound DmATL are displayed in Figure 5a and distinguished by different shades of purple:^38^ the G domain, which binds and hydrolyses GTP; the three-helix-bundle (3HB) domain; the two transmembrane (TM) domains; and the cytosolic tail (CT) domain.^38^ The fusion of two adjacent membranes, separated by an intermediate water layer, involves the *trans*-dimerization of two atlastin proteins across this water layer, as schematically shown in Figure 5b. The fusion process is believed to start with initial contacts between the G domains of the two atlastins (Figure 5b, upper cartoon), both of which are loaded with GTP. After this initial dimerization, the protein dimer is thought to undergo a conformational transition, the so-called crossover dimerization, in which the two G domains swap their relative positions, and the two three-helix-bundle (3HB) domains come into contact as well (Figure 5b, lower cartoon), where this conformational transition occurs after the cleavage of GTP to guanosine diphosphate (GDP) and inorganic phosphate (P_*i*_). The atlastin conformations displayed in Figure 5 are based on a couple of crystal structures^39,68,69^ and on the analysis of chemical kinetics.^38,39,70,71^

Because the dimerization process binds two identical protein monomers, one should expect that the associated process of GTP hydrolysis can also occur for two atlastins that are anchored to the same membrane, as schematically shown in Figure 5c. The latter process of *cis*-dimerization coupled to GTP hydrolysis has indeed been observed in experiments on supported lipid bilayers,^38^ but the significance and consequences of this latter type of dimerization are not understood.

#### Large-Scale Membrane Tension from *cis*-Dimerization

In order to elucidate the morphological puzzles of the ER as described above, we now view GTP hydrolysis by a *cis*-dimer (Figure 5c) as a local “hot spot” that moves the adjacent membrane patch out of equilibrium, thereby changing the spectrum of membrane undulations and enhancing these undulations on small length scales (Figure 3b–d). Based on the model for active *cis*-dimerization in Figure 5c, we obtain the following behavior of the “hot spots”. First, the *cis*-dimer is loaded with two GTP molecules, which are subsequently cleaved to GDP and inorganic phosphate, thereby changing its conformation from an extended one with widely separated transmembrane domains (Figure 5c, upper cartoon) to a more compact one in which the transmembrane domains are closer together (Figure 5c, lower cartoon). Such a conformational change of the *cis*-dimer will change the local bending moment that acts on the adjacent membrane patch. When the two inorganic phosphates and the two GDP molecules have been released, the *cis*-dimer will dissociate into two monomers, each of which will also generate a local bending moment. In this way, the “hot spot” will locally modify the membrane curvature and lead to local membrane bumps, which pull membrane area out from the surrounding membrane.

#### Membrane Area Pulled out by *cis*-Dimers

For the in vitro tubes in Figure 1c, the density of membrane-bound atlastin was about 1 protein per (20 nm)^2^. Assuming a comparable density for the in vivo tubes in Figure 3b–d, the TS1 tube segment in this figure (white dotted rectangle in Figure 3b) contains about 4000 protein monomers or 2000 *cis*-dimers. In order for these dimers to prevent the fragmentation of the tubes, they should increase the effective tension from Σ = Σ_1_ in eq 2 to Σ = Σ_2_ in eq 3. The corresponding excess area  pulled out by the dimers is then equal to , which corresponds to about 5% of the tube area *A*_0_ as shown in the Methods section.

The area *A*_0_ of the TS1 segment in Figure 3b–d is about 1.6 μm^2^, which implies that this segment does not rupture if the total membrane area pulled out by the 2000 dimers is about 0.05 × 1.6 μm^2^ or 0.08 μm^2^. Each dimer will then pull out an area of about 0.08 μm^2^/2000 = 40 nm^2^. This area should be compared with the spatial dimensions of the atlastin protein. The cytosolic domain of the atlastin monomer in Figure 5a consists of 415 residues, which form the G domain, a short flexible linker of a few residues, the 3HB domain, and another flexible linker of 14 residues, which connects the cytosolic domain to the adjacent TM domain. The 3HB bundle has a linear extension of about 4 nm, and the G domain has a diameter of about 4.5 nm. For the dimer conformation in the upper cartoon of Figure 5c, the separation of the two anchor segments is about 14 nm, whereas it is about 3 nm in the lower cartoon.^71^

The membrane segments that are locally affected by these dimer conformations depend on the flexibility of the two linkers between the two 3HB domains and the adjacent TM domains. For a flexible linker, the dimer can directly interact with the membrane. The dimer in the lower cartoon will then affect a membrane area of about π(8 nm)^2^ or 200 nm^2^. In addition, the four transmembrane domains of the dimer form two wedges in the membrane. For the dimer conformation in the lower cartoon of Figure 5c, these two wedges are likely to generate a large local curvature that bends the membrane away from the dimer. For the dimer conformation in the upper cartoon of Figure 5c, effective repulsive interactions between the adjacent membrane segment and the dimer may further enlarge the membrane segment affected by the dimer. Taken together, the above estimates indicate that the effective membrane tension generated by the *cis*-dimers can pull out enough excess area from the peristaltic shape fluctuations to prevent the fragmentation of the membrane tubes.

### Outlook on Future Experiments

#### Reconstitution of Membrane Proteins in Giant Vesicles

During the last two decades, giant unilamellar vesicles (GUVs) have been increasingly used as versatile research tools for basic membrane science, bioengineering, and synthetic biology. Thus, a variety of methods have been developed to prepare these giant vesicles and to characterize their physicochemical properties.^2,3^ In order to elucidate the behavior of the ER membranes, one would like to create GUVs doped with GTP-hydrolyzing proteins, such as atlastin or analogous membrane proteins. As a first step, one needs to express and purify the membrane proteins and then stabilize them via an appropriate buffer or via proteoliposomes.^25,33,35,37,38^ Furthermore, for other types of membrane proteins, a number of protocols have been described,^1,72−79^ by which one can insert the proteins and proteoliposomes into GUVs with a preferred orientation. However, for atlastin and related membrane proteins, a feasible protocol has not been reported thus far and remains to be developed.

It will be desirable to grow GUVs that contain only the membrane protein DmATL as well as GUVs with two membrane proteins such as the GTP-hydrolyzing protein Sey1p and the curvature-generating protein Yop1p.^25,36^ The latter combination was used to create the reticular network in Figure 1b, whereas the network in Figure 1c was based on DmATL alone. Combining two membrane proteins, one for curvature generation and one for homotypic fusion, has the advantage that one can control these two processes independently, at least to some extent, by varying the mole fractions of the two proteins in the GUV membranes. After the membrane proteins have been incorporated into the GUVs, one can use a variety of experimental methods to study the behavior of these GUVs and the dependence of this behavior on the GTP concentration.

#### GTP-Dependence of Freely Suspended GUVs

One relatively simple experiment is to determine the undulation spectrum of freely suspended and quasispherical GUVs,^80−82^ both in the absence and in the presence of GTP. The presence of GTP should lead to a significant change of this spectrum, a change that becomes more pronounced for higher GTP concentration and for larger densities of the membrane protein. As explained above, the formation of *cis*-dimers is expected to increase the large-wavenumber undulations and to decrease the small-wavenumber undulations, thereby generating an effective membrane tension on large scales. A different behavior has been observed for GUVs containing active bacteriorhodopsin pumps, which increased the undulations for all wavenumbers.^83^ Such an overall shift of the spectrum implies that the total membrane area is increased by the activity of the membrane pumps as follows from the systematic theoretical analysis in ref (84).

**Figure 6:**
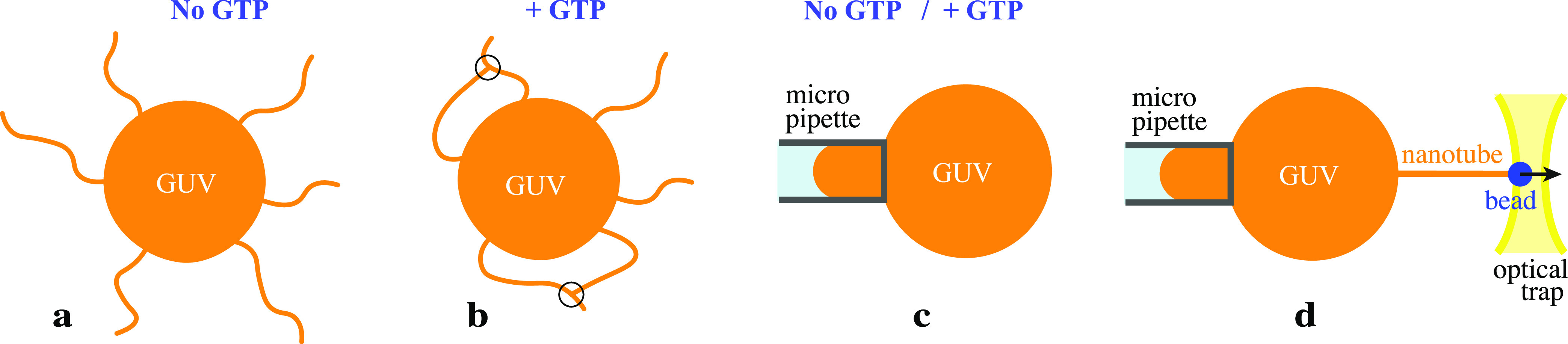
Membrane nanotubes connected to GUVs (orange) doped with membrane proteins: (a) Spontaneous tubulation of a GUV arising from protein-induced curvature generation in the absence of GTP. (b) When these proteins are able to hydrolyze GTP, the addition of GTP will change the morphology of the GUV and, in particular, lead to the formation of some three-way junctions (black circles). (c) Micropipette aspiration of a GUV in the absence and presence of GTP to measure the dependence of membrane tension on GTP concentration. (d) Tubule pulled from an aspirated GUV by an optical trap. When tube pulling is combined with superresolution microscopy,^88^ one should be able to observe the active membrane undulations of the tubule.

If the membrane protein has been inserted into the GUV membrane with a preferred orientation, the membranes are asymmetric and undergo spontaneous tubulation when the GUV is osmotically deflated; see Figure 6a. Such a spontaneous tubulation has been previously demonstrated for His-tagged proteins bound to the outer leaflet of the GUV membranes^65^ and for GUVs exposed to asymmetric polymer^85^ and sugar^86^ solutions. When GTP is added, two tubes that come into close contact may undergo fusion and form membrane junctions, as in Figure 6b. In this way, the GTP concentration can be used as a control parameter to modify the GUV morphology. To facilitate the detection of fusion events, one can use two GUV populations labeled with different fluorescent dyes.^87^ In addition to the fusion of nanotubes, a variation of the GTP concentration will also lead to shape changes of the mother vesicle because the GTP-dependent *cis*-dimerization of the proteins will also modify the spontaneous curvature.

#### GTP-Dependence of Membrane Tension

A widely used method to measure membrane tension in a quantitative manner is provided by micropipette aspiration, which has been used for a variety of living cells^89−92^ as well as for GUVs.^93,94^ The aspiration method can be combined with magnetic^95^ or optical^88,96,97^ tweezers to pull membrane nanotubes from the cells and from the giant vesicles. By modification of the externally applied forces caused by the magnetic or optical tweezers, one can directly control the tension within the cell and vesicle membranes.

In the present case, it will be useful to first aspirate the GUVs by micropipettes *prior* to deflation, i.e., in the absence of spontaneously formed nanotubes, and to measure the membrane tension as a function of GTP concentration; see Figure 6c. In addition, the spontaneous curvature can be determined by the aspiration of tubulated GUVs^86^ or by pulling tubes from a GUV as in Figure 6d, as previously shown for membrane systems close to equilibrium.^88,97,98^ When tube pulling is combined with super-resolution light microscopy such as SIM^54,55^ or STED,^99,100^ the shape of the nanotubes and their GTP-dependent undulations should become detectable.

#### Preliminary Results on the Reconstitution of Atlastin in GUV Membranes

**Figure 7:**
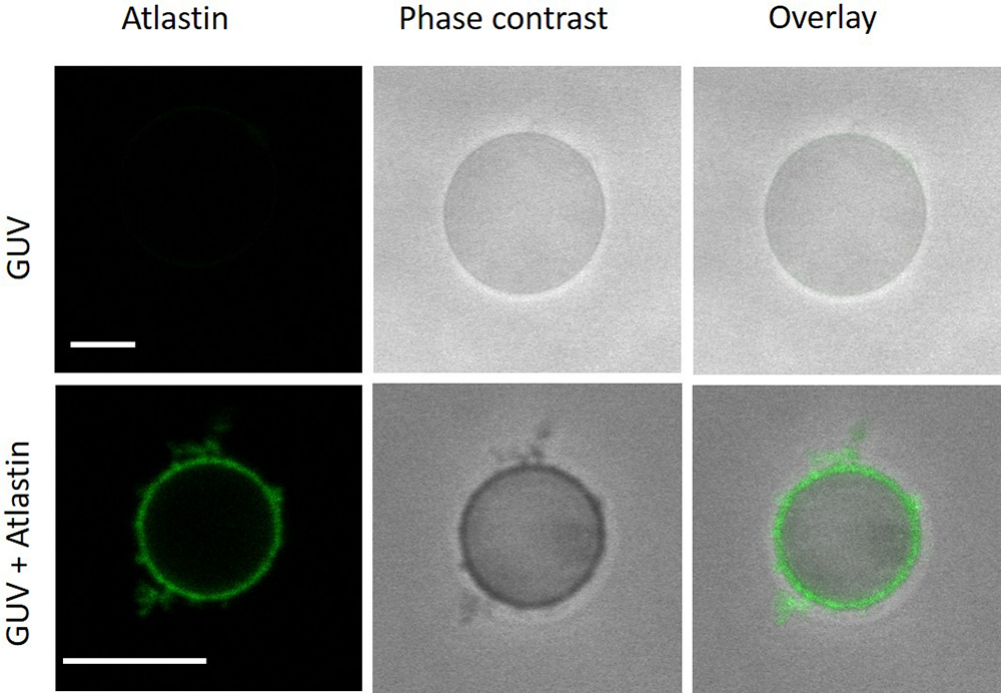
Incorporation of atlastin (DmATL) into GUV membranes. GUVs are prepared by PVA-assisted swelling with the lipid composition of POPC, POPG, and Chol in a molar ratio of 7:1:2. The vesicle solution is incubated with fluorescently labeled atlastin (green), which was inserted into the membrane in the presence of the detergent Triton X-100. The detergent is adsorbed and removed using Bio-Beads. The images display some atlastin-containing membrane protrusions. Further details of the experimental protocol are described in the Methods section. Both scale bars: 10 μm.

The first step of the experimental approach outlined in the previous paragraphs is to incorporate membrane protein DmATL into GUV membranes. We now report preliminary experimental results that provide direct evidence of such an incorporation. The GUVs are incubated with DmATL, which is fluorescently labeled using Oregon Green. The protein buffer contains detergent Triton X-100, which facilitates the incorporation of atlastin into the membranes. After removal of the detergent by Bio-Beads, we obtained proteo-GUVs with the DmATL protein incorporated in the GUV membranes. One example of such a proteo-GUV is depicted in Figure 7. Further details of our preparation protocol are described in the Methods section.

The fluorophore attached to the DmATL protein allowed us to directly detect the membrane-bound atlastin in the GUVs. The fluorophore may, however, impede the hydrolysis activity of the protein. Thus, in order to study this activity, we primarily use unlabeled atlastin in the next experiments.

## Summary and Conclusion

This paper addressed several experimental puzzles of the ER morphology: the irregular polygonal geometry of the tubular network with three-way junctions and contact angles close to 120° (Figure 1); the different shapes of nanotubes and junctions as obtained by electron microscopy and structured illumination microscopy (Figure 3); as well as the observations that the maintenance of the reticular network requires both GTP and GTP-hydrolyzing membrane proteins because the nanotubes undergo fragmentation when the biosynthesis of the GTP-hydrolyzing proteins is downregulated^33^ or the supply of GTP is interrupted.^25^

To explain these morphological puzzles, several theoretical concepts are introduced here. First, Steiner minimal trees as studied in mathematical graph theory^48,49,51,52^ are used to show that the irregular polygonal geometry with contact angles close to 120° provides strong evidence for a significant membrane tension within the ER membrane (Figure 2). Second, the smoothly curved shapes of triunduloids, which represent constant-mean-curvature shapes from differential geometry, are viewed as plausible models for the observed three-way junctions of the nanotubular networks (Figure 4d–f). These triunduloids have a low bending energy when their mean curvature is close to the spontaneous curvature of the ER membrane. Third, the curvature elasticity of the ER membranes implies that unduloids and triunduloids with closed membrane necks (Figure 4a,d) are most likely to undergo fission, which leads to tube fragmentation and to the destruction of the nanotubular networks as observed experimentally, both in vivo and in vitro, when the number of GTP-hydrolyzing proteins is reduced or the supply of GTP is interrupted. Therefore, in order to prevent such a fragmentation and the concomitant destruction of the networks, the undulating tubules and junctions should close their membrane necks only transiently for relatively short periods of time (Figure 3b-d).

Finally, it is argued that all experimental puzzles considered here can be resolved by the view that the GTP hydrolysis by *cis*-dimers on molecular scales generates an effective membrane tension on mesoscopic scales. Thus, according to this view, GTP hydrolysis by *cis*-dimerization of membrane proteins changes the spectrum of membrane undulations by enhancing the short-wavelength undulations, as in Figure 3b–d, and by generating an effective membrane tension for the long-wavelength undulations. This effective tension has two important consequences. First, it provides the membrane-elastic mechanism for the irregular polygonal geometry of the reticular networks with contact angles close to 120°. Second, the reduction of the available membrane area on larger length scales suppresses the formation of closed membrane necks (Figure 4a,d) and the subsequent fission of these necks.

So far, the hydrolysis of GTP by *cis*-dimerization of membrane proteins as depicted in Figure 5c seems to waste a lot of chemical free energy without any functional significance. However, when the active cis-dimerization process is viewed as a mechanism for the generation of membrane tension, this process becomes essential to maintain the integrity of the reticular network.

Once we have obtained a deeper understanding of the structure and dynamics of the reticular networks, we should be able to control and modify the network architecture in a systematic and quantitative manner. This architecture divides space into four different liquid (or fluid) compartments, as provided by the water channels enclosed by the nanotubes, the inner and outer leaflets of the tube membranes, and the exterior aqueous compartment. Particularly appealing objectives will be to vary the width of the nanotubes and the mesh size of the networks as determined by the spatial separation of the three-way junctions. These geometric features determine the area-to-volume ratio of the lipid–protein membranes, which may then be optimized to accommodate additional membrane-bound proteins and chemical reactions on these membranes. Indeed, the low density of membrane-bound proteins used previously to obtain the reconstituted reticular networks leaves ample space for other proteins to be anchored at the nanotube membranes. Likewise, it will also be useful to vary the network geometry in order to adjust the interior volume of the nanotubes, which represent a complex nanofluidic compartment with flexible walls as provided by the inner leaflets of the membranes. Eventually, it may even become possible to add membrane proteins that mediate and control transport between the different subcompartments of the network architecture.

## Methods

### Peristaltic Shape Fluctuations of Membrane Nanotubes

#### Unstable Modes and Effective Membrane Tension

We start from a membrane nanotube that has the shape of a cylinder with length *L* and radius *r*_0_. As in Figure 4c, the mean curvature of the cylinder, *M* = 1/(2*r*_0_), is taken to be equal to the spontaneous curvature *m*. Peristaltic deformations of this tube preserve the cylindrical symmetry, which implies that the corresponding deformation field depends only on the coordinate *z* along the cylinder axis and is independent of the azimuth angle. The peristaltic deformations, *l* = *l*(*z*), of the tube are decomposed into Fourier modes according to
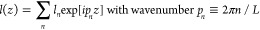
4where the sum runs over the integers *n* from – *n*_max_ ≤ *n* ≤ *n*_max_. The largest integer *n* = *n*_max_ corresponds to the high wavenumber cutoff *p*_max_ ≡ 2*πn*_max_/*L* = 2π/_me_ where _me_ is the thickness of the bilayer membrane. The Fourier modes *l*_*n*_ in eq 4 are given by
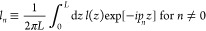
5and *l*_0_ ≡ 0 for *n* = 0. The modes *l*_*n*_ are governed by the statistical weight  with the configuration energy , where the bending energy  is proportional to the membrane’s bending rigidity κ and the second energy term  involves the effective tension, Σ, which determines the area reservoir accessible to the membrane segment as well as the excess area Δ*A*{*l*} stored in the shape fluctuations.

In terms of the Fourier modes *l*_*n*_, the bending energy has the form^101,102^
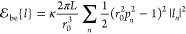
6for mean curvature *M* = 1/(2*r*_0_) = *m* where *m* denotes the spontaneous curvature as before. The effective tension term is given by
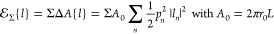
7

An analogous tension term has been previously considered for planar membrane segments^103^ and for quasispherical vesicles.^80,104^

The bending energy term in eq 6 includes the peristaltic mode  with vanishing bending energy . This zero-energy mode is characterized by the wavenumber
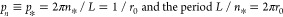
8

It turns out that this period is identical to the limiting period of an unduloid that approaches the cylindrical shape. Thus, the zero-energy mode corresponds to the deformation of the cylinder into an unduloid, which is, however, suppressed by the effective tension Σ.

Using the statistical weight , we obtain the mean-squared mode amplitudes
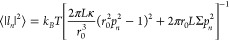
9which define the fluctuation spectrum of the peristaltic modes. For the zero-energy mode , which represents the most unstable mode, the first term in eq 9 vanishes, which leads to eq 1 in the main text.

#### Excess Area Stored in Peristaltic Modes

The excess area stored in the peristaltic modes depends on the magnitude of the effective membrane tension Σ. Using the statistical weight for the peristaltic modes as introduced in the previous paragraph and the area *A*_0_ = 2*πr*_0_*L* of the undeformed cylinder, the average excess area  stored in the peristaltic modes is found to be
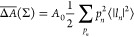
10where the Σ-dependence is contained in the mean-square amplitudes ⟨|*l*_*n*_|^2^⟩. Inserting the expression for these amplitudes as given by eq 9 and transforming the discrete sum over *p*_*n*_ into an integral, one obtains the asymptotic equality
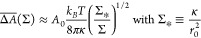
11for small Σ ≪ Σ_*_. For κ = 10^–19^ J and *r*_0_ = 100 nm, the tension scale Σ_*_ = 10^–2^ mN/m. Using eq 11 to compute the excess area  for the tension Σ_1_ as estimated for tube fragmentation, see eq 2 in the main text, we obtain the average excess area , corresponding to 10% of the undeformed tube area *A*_0_. Furthermore, for the tension Σ_2_ in eq 3, the average excess area has the value . Therefore, when we increase the effective tension from Σ_1_ to Σ_2_, we reduce the excess area stored in the shape fluctuations from 10 to 5% of the tube area *A*_0_, thereby preventing fragmentation of the tube.

### Experimental Protocol for Atlastin Reconstitution in GUVs

#### Protein Purification and Labeling

The DmATL protein was expressed and purified according to the protocol in ref (35). The final buffer of purified atlastin consisted of 25 mM HEPES at pH 7.5, 100 mM KCl, 10% (v/v) glycerol, 1% Triton X-100, 2 mM DTE, and 10 mM glutathione reduced. The protein was labeled with Oregon Green 488 succinimidyl ester, purchased from Thermo Fischer. The protein and dye were mixed in the molar ratio 1:1 and incubated at 4 °C overnight. The free dye was removed using a dialysis membrane with a cutoff of 7 kDa.

#### Preparation of GUVs

The GUVs were made by poly(vinyl alcohol) (PVA)-assisted swelling of lipid bilayers.^105^ The PVA with a molecular weight of *M*_*w*_ = 145 kDa was purchased from Merck, Germany. A solution of 40 mg/mL of PVA was prepared by adding PVA to water. The solution was heated to 90 °C and mixed using a thermomixer. A 20 μL aliquot of this PVA solution was deposited on a glass slide, spread using a pipet tip, and dried on a hot plate at 60 °C for 30 min. The lipid bilayers were prepared from chloroform stock solutions of three lipids, 1-palmitoyl-2-oleoyl-*sn*-glycero-3-phosphocholine (POPC), 1-palmitoyl-2-oleoyl-*sn*-glycero-3-phospho-(1′-*rac*-glycerol) (POPG), and cholesterol (Chol), purchased from Avanti Polar Lipids (Alabaster, AL). A volume of 8 μL with a total lipid concentration of 2 mM (POPC:POPG:Chol in the molar ratio of 7:1:2) was spread uniformly on the PVA bed. The chloroform was evaporated by drying under a stream of nitrogen. A small chamber was formed around the dried lipid using a Teflon spacer, another glass slide, and fold-back clips. The chamber was filled with the atlastin buffer (25 mM HEPES pH 7.4, 100 mM KCl, 10% glycerol, 1 mM EDTA, 2 mM β-mercaptoethanol; all chemicals purchased from Thermo Scientific). The vesicles were harvested after 30 min.

#### Incorporation of Atlastin in GUVs

The vesicle solution was incubated with the Oregon Green labeled DmATL, following the protocol described in ref (74). The protein buffer contained detergent Triton X-100. This detergent destabilized the GUV membrane and assisted in the incorporation of atlastin. This mixture was incubated for 15 min at room temperature. The excess detergent was removed by incubating the vesicle solution with Bio-Beads SM-2 Resin (Bio-Rad) for 2 h at 4 °C. The beads used for the detergent removal were replaced every 30 min by fresh Bio-Beads. After this procedure, atlastin was incorporated into the GUV membranes as demonstrated by their green fluorescence. One example for such a proteo-GUV is shown in Figure 7.

#### Imaging of GUVs

All samples were imaged using a Leica SP5 point scanning confocal microscope (Wetzlar, Germany). The GUVs were settled on the coverslide, using a low-density imaging buffer (70 mM HEPES pH 7.4, 340 mM KCl, 8% glycerol, 1 mM EDTA, 2 mM β-mercaptoethanol). The osmolarity of this imaging buffer was adjusted with an osmometer (Osmomat 3000, Gonotec GmbH, Germany) to match the osmolarity of the atlastin buffer mentioned above. The solution containing the GUVs or the proteo-GUVs was mixed with the imaging buffer in a volume ratio of 2:1. The imaging buffer was just used to settle the proteo-GUVs on the coverslide and played no other functional role.
